# Expression of *HOXA11 *in the mid-luteal endometrium from women with endometriosis-associated infertility

**DOI:** 10.1186/1477-7827-10-1

**Published:** 2012-01-10

**Authors:** Malgorzata Szczepańska, Przemyslaw Wirstlein, Jana Skrzypczak, Paweł P Jagodziński

**Affiliations:** 1 Department of Obstetrics, Gynecology and Gynecological Oncology, Division of Reproduction, Poznan Medical University of Sciences Poland; 2 Department of Biochemistry and Molecular Biology, Poznan Medical University of Sciences Poland

## Abstract

**Background:**

A decrease in *HOXA11 *expression in eutopic mid-secretory endometrium has been found in women with endometriosis-associated infertility.

**Methods:**

Using Real-time quantitative PCR (RQ-PCR) and western blotting analysis we studied the HOXA11 transcript and protein levels in mid-luteal eutopic endometrium from eighteen infertile women with minimal endometriosis, sixteen healthy fertile women and sixteen infertile women with fallopian tubal occlusion from the Polish population. We also evaluated transcript levels of DNA methyltransferases DNMT1, DNMT3A and DNMT3B in these groups of women.

**Results:**

There were significantly lower levels of HOXA11 transcripts (p = 0.003, p = 0.041) and protein (p = 0.004, p = 0.001) in women with endometriosis as compared to fertile women and infertile women with tubal occlusion. Moreover, we found significantly higher methylation levels of the CpG region in the first exon of *HOXA11 *in infertile women with endometriosis compared with fertile women (p < 0.001) and infertile women with tubal occlusion (p < 0.001). We also observed significantly increased levels of DNMT3A transcript in women with endometriosis than fertile women (p = 0.044) and infertile women with tubal occlusion (p = 0.047). However, we did not observe significant differences in DNMT1 and DNMT3B transcript levels between these investigated groups of women.

**Conclusions:**

We confirmed that reduced *HOXA11 *expression may contribute to endometriosis-associated infertility. Moreover, we found that DNA hypermethylation can be one of the possible molecular mechanisms causing a decrease in *HOXA11 *expression in the eutopic mid-secretory endometrium in infertile women with endometriosis.

## Background

Endometriosis is a gynecological disease that is characterized by occurrence of tissue similar to the lining of the uterus elsewhere in the body [1,2]. Endometriosis lesions can be located in the cavity and walls of the pelvis, on the ovaries, the fallopian tubes, the rectal-vaginal septum, and other body sites [1,2]. This disease is often accompanied by pelvic pain, inflammation and results in infertility in 30 to 50% of the affected women [3-5].

It has been suggested that endometriosis-associated infertility may be due to disorders of folliculogenesis, decreased fertilization, defective implantation, and reduced oocyte quality with low capacity of blastocyst implantation [6,7]. Estrogen and progesterone are responsible for the regulation of numerous genes' expression during the follicular and luteal phases of the menstrual cycle [8]. In women with endometriosis, progesterone is not able to induce several genes' expression during the window of implantation as compared to women without endometriosis. This defective response to progesterone may cause hostile conditions for blastocyst implantation in women with endometriosis [9-11].

The occurrence of endometriosis can be linked to some genetic factors, immunological disorders, defective estrogen metabolism, and exposure to environmental contamination and toxins [12-15]. However, the etiopathogenesis and pathophysiology of subfertility in women with endometriosis is still elusive [2].

Endometriosis has been recognized as a disease accompanied by aberrant methylation and expression of *steroidogenic factor-1 *(*SF-1*), *estrogen receptor 2 (ESR2)*, *progesterone receptor (PR-B) *and *HOXA10 *genes in the eutopic endometrium of women with endometriosis [16-19]. The deficient expression of *HOXA10 *and *HOXA11 *in infertile women with endometriosis and in animal models has been demonstrated [16,20-23]. However, it is still unclear whether the observed decreased expression of the *HOXA11 *gene can be related to its hypermethylation in endometriosis-associated infertility. Therefore we studied the effect of DNA regulatory sequences' methylation on the HOXA11 transcript and protein levels in eighteen infertile women with minimal endometriosis, sixteen fertile women and sixteen infertile women with fallopian tubal occlusion from a Polish cohort. In these groups of women, we also evaluated transcript levels of DNA methyltransferases DNMT1, DNMT3A and DNMT3B.

## Methods

### Patients and controls

Since advanced endometriosis may lead to anatomical distortions and adhesions resulting in infertility, we focused our study on infertility associated with minimal endometriosis. Data for women with infertility-associated endometriosis, fertile women and infertile patients with fallopian tubal occlusion were randomly collected in the Gynecologic and Obstetrical University Hospital, Division of Reproduction in Poznan, Poland (Table 1). Women with endometriosis and fertile women were examined for the cause of infertility, suspected pelvic endometriosis, or chronic pelvic pain. Then they were divided into eighteen infertile women with minimal endometriosis and sixteen fertile women. Minimal endometriosis in infertile women was diagnosed based on visualization of endometriotic lesions and histopathologic criteria (Table 1). The stage of endometriosis was evaluated according to the revised classification of the American Society for Reproductive Medicine [24]. The studied women with endometriosis displayed no anatomical changes in the reproductive tract. The women with endometriosis and with fallopian tubal occlusion exhibited regular menses and a minimum 1 year of infertility with a current desire for conception, and no contribution of male factor infertility. The fertile women assigned to the control group exhibited chronic pelvic pain without any pelvic abnormalities determined by laparoscopy. The fertile women were diagnosed as having varicose veins in the pelvic floor but no signs of past or present inflammation. These fertile women had at least one child born no later than 2 years before laparoscopy, regular menses, and no anatomical changes in the reproductive tract (Table 1). The second control group included the women with fallopian tubal occlusion diagnosed based on hysterosalpingography and subsequently verified by methyl blue administration to fallopian tubes during laparoscopy [25]. Furthermore, hysteroscopy and pipelle biopsy from women with endometriosis, and fertile women and infertile women with tubal occlusion were respectively employed for histopathologic evaluation to exclude individuals with pathological endometrium. All participating individuals had not used oral contraception, hormonal therapy, or an intrauterine device for half a year prior to the endometrial biopsy. Fertile women and infertile women with tubal occlusion were matched by age to the patients with endometriosis and all individuals were Caucasian from the same region of Poland (Table 1). Written informed agreement was obtained from all participating individuals. The procedures of the study were approved by the Local Ethical Committee of Poznań University of Medical Sciences. All biopsy specimens were collected during the middle secretory phase based on the endometrial dating criteria of Noyes *et al*. [26]. Sample of eutopic endometrial tissue from patients and controls was respectively obtained by pipelle or hysteroscopic biopsy during the implantation window, i.e. 7-9 days after ultrasound-confirmed ovulation. The eutopic endometrium samples were then used for total RNA, protein, and DNA isolation.

**Table 1:** Clinical characteristics of infertile women with minimal endometriosis, fertile women and infertile women with tubal occlusion.

Characteristics	Endometriosis	Fertile women	Tubal occlusion
Number of patients	18	16	16
Age (years)	31 (24-38)^a^	31 (25-37)^a^	32 (25-39)^a^
Parity	NA	2 (1-3) ^a^	NA
Duration of infertility (Years)	3 (1-4)	NA	3 (1-4)
rASRM stage^b^	Stage I (n = 8) Stage II (n = 10)	NA	NA

Median (Range)^a^, revised American Society for Reproductive Medicine classification (rASRM)^b ^[24], NA-not applicable.

### Antibodies

Goat polyclonal (Gp) anti-HOXA11 antibodies (Ab) (C-16, 200 μg/1.0 ml), donkey anti-goat horseradish peroxidase (HRP)-conjugated Ab (200 μg/0.5 ml), and anti-actin HRP-conjugated Ab (clone I-19, 200 μg/1.0 ml were provided by Santa Cruz Biotechnology (Santa Cruz, CA).

### Real-time quantitative PCR (RQ-PCR) analysis of HOXA11 and DNA methyltransferase transcript levels

Total RNA was isolated by RNeasy Protect Mini Kit Qiagen (Hilden, Germany). RNA samples were treated with DNase I from DNase Set Qiagen (Hilden, Germany) and quantified. A volume of 0.5 μg RNA was reverse-transcribed into cDNA using QuantiTect Reverse Transcription Kit and oligo-dT primers from Qiagen (Hilden, Germany). The efficiency of reverse transcription was controlled by a series of RNA dilution-reverse-transcribed and comparison of RQ-PCR Ct differences for cDNA samples [27].

RQ-PCR was conducted in a Corbett Research Rotor-Gene 3000 thermocycler (Mortlake, Australia). Target cDNA was quantified using relative quantification method employing a calibrator. The calibrator was prepared as a cDNA mix from all patients and control samples and consecutive dilutions were used to create a standard curve as provided in Relative Quantification Manual Roche Diagnostics GmbH (Mannheim, Germany). The quantity of HOXA11, DNMT1, DNMT3A and DNMT3B transcript in each sample was standardized by human glyceraldehyde 3-phosphate dehydrogenase (GAPDH) and β-actin (ACTB) transcript levels. For amplification, 60 ng cDNA solution was added to 18 μl (total 20 μl) of DyNAmo HS SYBR^® ^Green qPCR Kit from Finnzymes (Espoo, Finland) and primers (Additional file 1, Table S1). One RNA sample of each preparation was processed without the reverse transcription (RT)-reaction to provide a negative control in subsequent PCR. The quantity of HOXA11 transcripts in each sample was standardized by GAPDH and ACTB cDNA. Each sample was determined in triplicate and the HOXA11, DNMT1, DNMT3A and DNMT3B mRNA levels were expressed as the multiplicity of these cDNA concentrations in the calibrator.

### Sodium dodecyl sulfate-polyacrylamide gel electrophoresis (SDS-PAGE) and western blot analysis

Tissue samples were minced in liquid nitrogen followed by lysis in RIPA buffer. Next, 30 μg of protein were resuspended in sample buffer and separated on 12% Tris-glycine gel using the SDS-PAGE system. Gel proteins were transferred to PVDF membrane, which was blocked with 5% milk in Tris-buffered saline/Tween. Immunodetection was performed with Gp anti-HOXA11 Ab (C-16, 1:500 dilution, 4.0 μg) followed by incubation with donkey anti-goat horseradish peroxidase (HRP)-conjugated Ab (1:3000 dilution, 1.3 μg). The membranes were then stripped and incubated with anti-β-actin HRP-conjugated Ab (clone I-19, 1:3000 dilution, 0.66 μg) to ensure equal protein loading of the lanes. Bands were revealed using ECL kit and Hyperfilm ECL Amersham (Piscataway, NJ). The quantities of western blot-detected HOXA11 and β-actin proteins were determined based on the band optical density. The band densitometry readings were normalized to β-actin loading control to calculate the HOXA11-to-β-actin optical density ratio.

### Sodium bisulfite DNA sequencing of cytosine-guanine dinucleotide (CpG) rich regions of the *HOXA11 *gene

Genomic DNA was isolated by the salting out method [28], and DNA cytosine bases were converted to uracil using the EZ DNA Methylation Kit™ procedure from Zymo Research Corporation (Orange, CA). The locations of CpG island in regions I, II, and III of the *HOXA11 *gene (Additional file 2, Figure S1) was determined based on two online programs [29,30].

The *HOXA11 *regions I, II, and III were amplified from the bisulfite-modified DNA by the three pairs of primers complementary to the bisulfite-DNA modified sequence (Additional files 1, Table S1; Additional file 2, Figure S1). PCR amplification was conducted by FastStart Taq DNA Polymerase from Roche Diagnostic GmbH (Mannheim, Germany). The PCR products were purified using Agarose Gel DNA Extraction Kit Roche (Mannheim, Germany) with subsequent cloning into pGEM-T Easy Vector System I Promega (Madison, WI) and transformation into TOPO10 *E. coli *strain cells. Plasmid DNA isolated from ten positive bacterial clones was used for commercial sequencing of the cloned fragment of DNA. The results of bisulphite sequencing were assessed and presented using BiQ analyzer software [31] and BDPC web server [32].

### Statistical analysis

Statistical analysis was conducted by Systat Software Inc (2006). SIGMASTAT (data analysis software system) version 3.5 [33]. Data groups were analyzed by Mann-Whitney Rank Test to evaluate if there was significance (P < 0.05) between the groups.

## Results

### Levels of HOXA11 transcript and protein in infertile women with endometriosis, fertile women and infertile women with tubal occlusion

**Figure 1:**
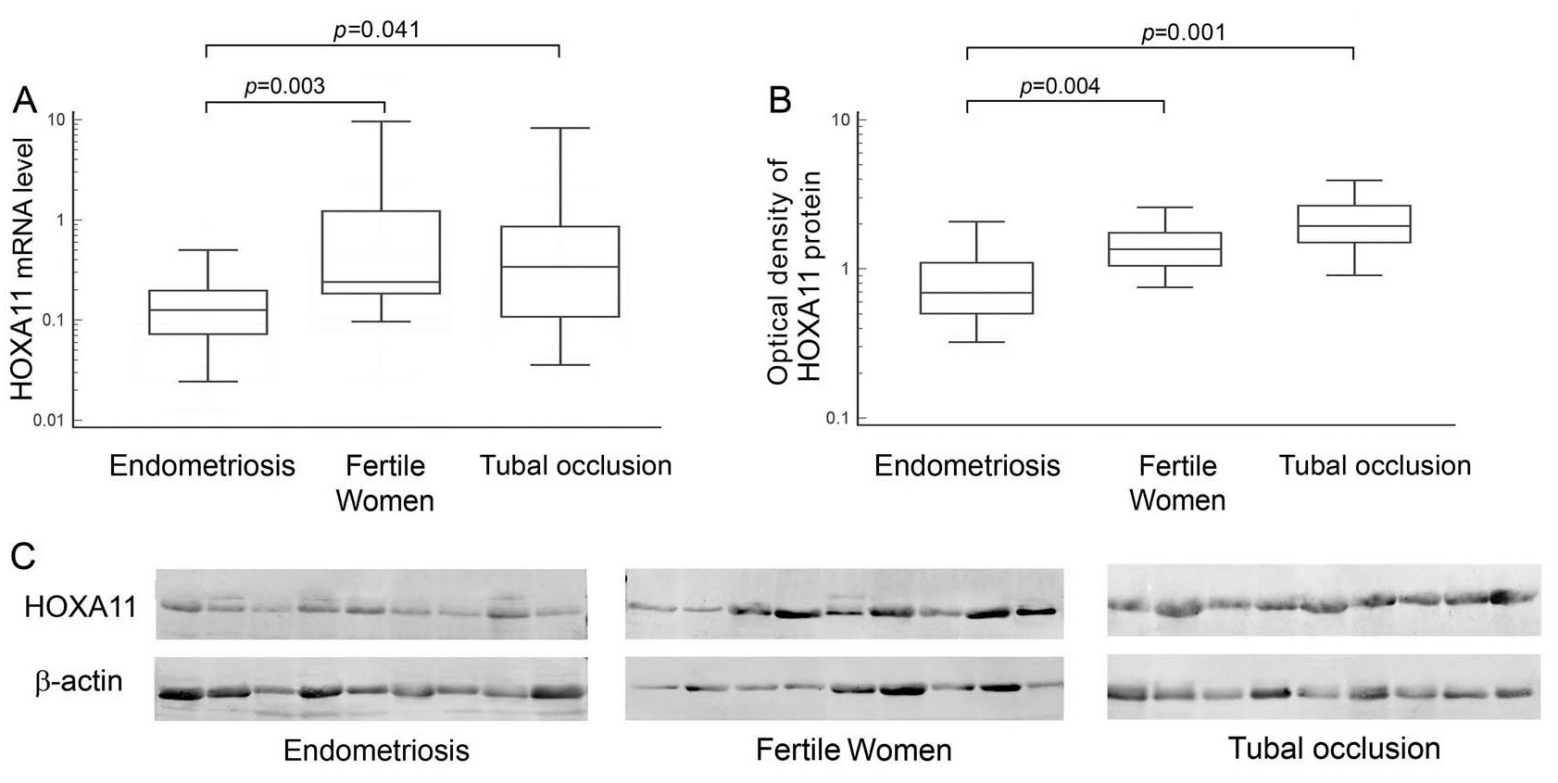
**HOXA11 transcript (A) and protein (B) levels and representative picture of western blot analysis of HOXA11 protein contents (C) in eutopic mid-luteal endometrium from infertile women with endometriosis, fertile women and infertile women with tubal occlusion**. The eutopic endometrium tissue from women with endometriosis, fertile women and infertile women with tubal occlusion was respectively obtained by pipelle or hysteroscopic biopsy during the implantation window, followed by total RNA and protein isolation. RNA was reverse-transcribed and *HOXA11 *cDNAs were investigated by RQ-PCR relative quantification analysis. To normalize the quantity of transcripts in each sample, HOXA11 mRNA levels were normalised to the amount of GAPDH and ACTB cDNA. The amounts of HOXA11 mRNA were expressed as the multiplicity of these cDNA copies in the calibrator. For western blot analysis, proteins were separated by SDS-PAGE and transferred to a PVDF membrane. This membrane was then incubated with Gp anti-HOXA11 Ab, followed by incubation with anti-goat HRP-conjugated Ab. To ensure equal protein loading of the lanes, the membranes were also incubated with anti-actin HRP-conjugated Ab. The amount of western blot-detected HOXA11 proteins was presented as the HOXA11-to-β-actin band optical density ratio. The boxes and the middle lines correspond to the values from the lower to upper quartiles and medians, respectively.

We used RQ-PCR and western blotting analysis to evaluate HOXA11 transcript and protein levels, respectively, in eutopic mid-secretory endometrium from infertile women with endometriosis, fertile women and women with tubal occlusion. We observed significantly lower levels of HOXA11 transcript in women with endometriosis as compared to fertile women (p = 0.003) and women with tubal occlusion (p = 0.041) (Table 2, Figure 1A). We also found significantly reduced HOXA11 protein levels in eutopic endometrium from infertile women with endometriosis than in fertile women (p = 0.004) and women with tubal occlusion (p = 0.001) (Table 2, Figures 1B and 1C).

**Table 2:** HOXA11 transcript and protein levels in eutopic mid-luteal endometrium from infertile women with endometriosis, fertile women and infertile women with tubal occlusion

Endometriosis		Fertile women		p^c^	Tubal occlusion		p^d^
Median (Range)	Mean (± SD)	Median (Range)	Mean (± SD)		Median (Range)	Mean (± SD)	
0.125 (0.0252- 0.469)^a^	0.155 ± 0.118^a^	0.246 (0.101- 8.669)^a^	1.449 ± 2.608^a^	0.003	0.322 (0.035- 7.266)^a^	0.922 ± 1.710^a^	0.041
0.694 (0.316- 3.772)^b^	1.023 ± 0.873^b^	1.385 (0.753-4.312)^b^	1.608 ± 0.889^b^	0.004	1.994 (0.837 - 5.859)^b^	1.570 ± 0.821^b^	0.001

^a ^The target mRNA levels were corrected to the amount of ACTB and GAPDH cDNA and expressed as multiplicity of these cDNA copies in the calibrator. ^b^The amount of Western blot-detected proteins was presented as the HOXA11-to-β-actin protein band optical density ratio. P values for ^c^endometriosis vs fertile women or ^d^endometriosis vs infertile women with tubal occlusion were assed by Mann-Whitney Rank Sum Test.

### DNMT1, DNMT3A and DNMT3B transcript levels in infertile women with endometriosis, fertile women and infertile women with tubal occlusion

**Figure 2:**
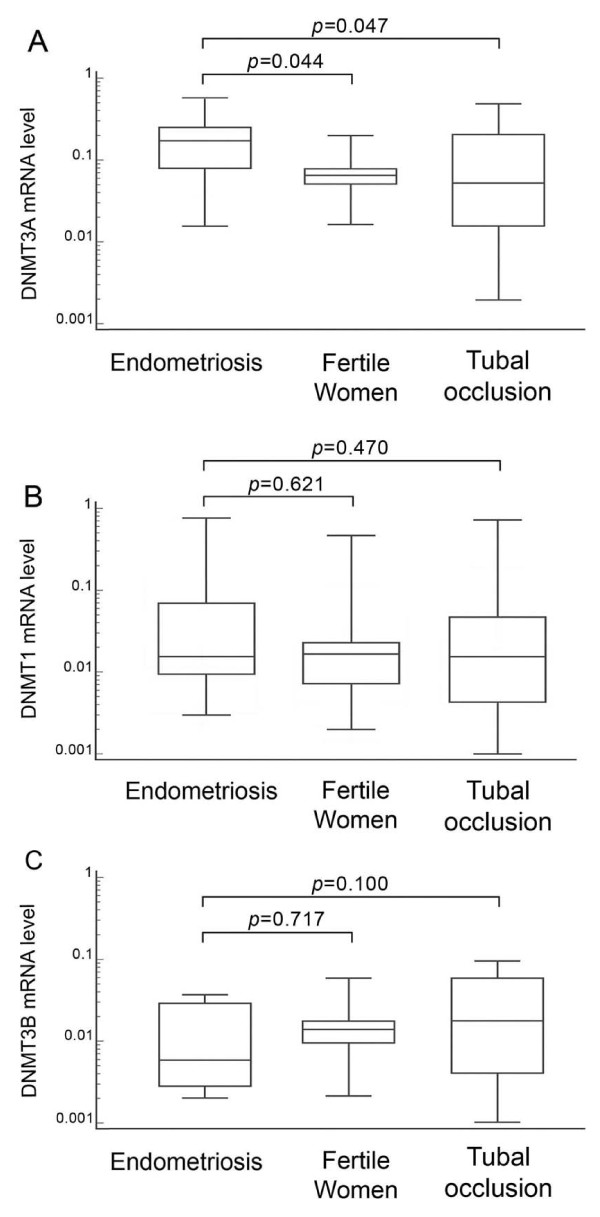
**DNMT1 (A), DNMT3A (B) and DNMT3B (C) transcript levels in eutopic mid-luteal endometrium from infertile women with endometriosis, fertile women and infertile women with tubal occlusion**. The eutopic endometrial tissue from women with endometriosis, fertile women and infertile women with tubal occlusion was respectively obtained by pipelle or hysteroscopic biopsy during the implantation window, followed by total RNA isolation. RNA was reverse-transcribed and DNMT1, DNMT3A and DNMT3B cDNAs were investigated by RQ-PCR relative quantification analysis. To normalize the quantity of transcripts in each sample, DNMTs mRNA levels were normalised to the amount of GAPDH and ACTB cDNA. The amounts of DNMTs mRNA were expressed as the multiplicity of these cDNA copies in the calibrator. The boxes and the middle lines correspond to the values from the lower to upper quartiles and medians, respectively.

RQ-PCR analysis showed significantly increased levels of DNMT3A transcript in eutopic mid-secretory endometrium from women with endometriosis compared to fertile women (p = 0.044) and women with tubal occlusion (p = 0.047) (Table 3 and Figure 2A). However, we did not observe significant differences in DNMT1 (p = 0.621, p = 0.470) and DNMT3B (p = 0.717, p = 0.100) transcript levels between the investigated groups (Table 3 and Figures 2B and 2C).

**Table 3:** DNMT1, DNMT3A and DNMT3B transcripts in eutopic mid-luteal endometrium from infertile women with endometriosis, fertile women and infertile women with tubal occlusion.

Gene	Endometriosis		Fertile women		p^a^	Tubal occlusion		p^b^
	Median (Range)	Mean (± SD)	Median (Range)	Mean (± SD)		Median (Range)	Mean (± SD)	
DNMT1	0.015(0.075 - 0.003)	0.094 ± 0.216	0.017(0.047- 0.003)	0.018 ± 0.013	0.621	0.015(0.071 - 0.001)	0.026 ± 0.025	0.470
DNMT3A	0.182(0.590 - 0.016)	0.203 ± 0.173	0.066(0.196- 0.017)	0.07 ± 0.052	0.044	0.042(0.508 - 0.002)	0.118 ± 0.173	0.047
DNMT3B	0.005(0.036 - 0.001)	0.014 ± 0.014	0.013(0.058- 0.002)	0.016 ± 0.016	0.717	0.019(0.1 - 0.001)	0.034 ± 0.034	0.100

The target mRNA levels were corrected to the amount of ACTB and cDNA and expressed as multiplicity of these cDNA copies in the calibrator. P values for ^a^endometriosis vs fertile women or ^b^endometriosis vs infertile women with tubal occlusion were assed by Mann-Whitney Rank Sum Test.

### DNA methylation levels of *HOXA11 *CpG rich regions in eutopic mid-luteal endometrium from infertile women with endometriosis, fertile women and infertile women with tubal occlusion

**Figure 3:**
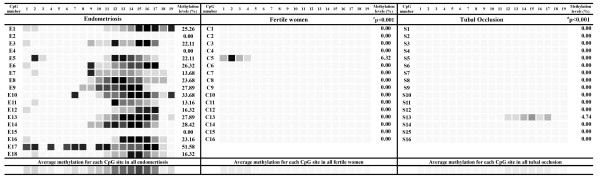
**Percentage of methylation of *HOXA11*, region II in infertile women with endometriosis (E1-18), fertile women (C1-16) and infertile women with tubal occlusion (S1-S16)**. Eutopic mid-luteal endometrium samples were used for genomic DNA isolation followed by bisulfite conversion of cytosine bases to uracil. The *HOXA11 *II region was then amplified by a pair of primers complementary to the bisulfite-DNA modified sequence (Aditional file 1, Table S1; Aditional file 2, Figure S1). The PCR products were purified with subsequent cloning into a plasmid vector. Plasmid DNA isolated from ten positive bacterial clones was used for commercial sequencing. The results of bisulphite sequencing were assessed and presented using BiQ analyzer software [31] and BDPC web server [32]^a^Mann-Whitney Rank Sum Test.

We performed sodium bisulfite DNA sequencing of *HOXA11 *regions I, II, and III (Additional file 2, Figure S1). There was no DNA methylation in *HOXA11 *regions I and III in all infertile women with endometriosis and all fertile women and women with tubal occlusion. However, we found significantly higher methylation levels of *HOXA11 *region in II the in eutopic mid-secretory endometrium obtained from infertile women with endometriosis as compared to material obtained from the fertile women (p < 0.001) and women with tubal occlusion (p < 0.001) (Figure 3). In the group of eighteen infertile women with endometriosis, we found fifteen (83.3%) individuals with methylation in *HOXA11 *region II (Figure 3). By contrast, in both fertile women and women with tubal occlusion we found one (6.2%) subject with DNA methylation in *HOXA11 *region II (Figure 3).

## Discussion

*Hoxa11 *belongs to the *Hox *gene family, which are genes that encode transcription factors expressed throughout embryonic development [34]. An equivalent of *Hoxa11 *has been found in the murine model as the *Abdominal-B-type homeobox *gene expressed in the limbs, kidney and stromal cells surrounding the Mullerian and Wolffian ducts [35]. Continued expression of *hox *genes has also been found in the female reproductive tract [36,37]. Mice that have either either *Hoxa11 *or *Hoxa10 *gene deletion are sterile, suggesting that these genes' products play an elementary role in endometrial growth, differentiation, receptivity, embryonic development, and female fertility [36,37].

Previously, we reaffirmed that DNA hypermethylation can be one of the mechanisms silencing *HOXA10 *expression in the mid-secretory endometrium in infertile women with endometriosis [38]. We subsequently decided to extend this study for *HOXA11 *in these patients.

In present study we confirmed that both HOXA11 mRNA and protein levels were significantly decreased in eutopic mid-luteal endometrium in infertile women with endometriosis as compared to fertile women. However, there were no correlations between HOXA11 transcript and protein levels to age, disease duration, and clinical characteristics of patients with endometriosis (results not shown).

*HOXA10 *and *HOXA11 *have displayed significant up-regulation in endometrial glands and stroma in humans during the mid-luteal phase at the period of implantation [39,40]. By contrast, women with endometriosis did not demonstrate an increase in the expression of these genes throughout the window of implantation [20,21]. The reduced expression of *HOXA11 *along with *HOXA10 *in the endometrium has been reported by Taylor *et al*. (1999), who suggested that this may result in infertility in patients with endometriosis [21]. Recently, Rackow *et al*. (2011) demonstrated a marked decrease in HOXA11 and HOXA10 mRNA levels in women with endometrial polyps with reduced pregnancy rates [41].

Our bisulfite DNA sequencing of *HOXA11 *CpG rich region II (Additional file 2, Figure S1) showed significantly increased levels of DNA methylation in eutopic mid-secretory endometrium from infertile women with minimal endometriosis as compared to fertile women. However, we did not find DNA methylation in *HOXA11 *CpG rich regions I and III in the same patients. Differential methylation in these regions might be due to distinct histone modifications and/or distinct interactions of nuclear proteins and non-coding RNAs to various chromatin conformations. These events might modulate DNA methyltransferase (DNMTs) accessibility to DNA, resulting in differentially methylated gene regions [42].

The hypermethylation of *HOXA10 *DNA regulatory sequences have been well documented to date in humans, and in murine and baboon endometriosis [16,22,43]. However, little is know about effect of *HOXA11 *gene methylation on its expression in infertile women with endometriosis. Recently, it has been demonstrated that *HOXA11 *DNA methylation is significantly associated with residual tumors after cytoreductive surgery and is a marker independently associated with poor outcome in ovarian cancer [44].

In humans, three CpG islands in the *HOXA11 *gene were localized: the first is 2408 bp upstream of exon 1, the second is mainly in exon 1, and the third is in the intron separating exons 1 and 2. (Additional file 2, Figure S1). The methylation of mammalian genomic DNA is carried out by DNMTs [45]. The role of some DNMTs in silencing *HOXA *gene transcription in eutopic endometrium in women with endometriosis has been reported [46]. We observed markedly increased levels of DNMT3A transcript in eutopic mid-secretory endometrium from women with endometriosis compared to fertile women. Our observations were on par with Wu *et al*. (2007), who also found significantly higher levels of DNMT3A in eutopic endometrium from infertile women with endometriosis as compared to controls [46].

Endometriosis has been considered as an epigenetic disease. Hypomethylation of *SF-1 *and *ESR2 *promoters may be responsible for increased estrogen action in women with endometriosis [18,19]. By contrast, a loss of progesterone response in women with endometriosis may be associated with hypermethylation of the *PR-B *promoter and a reduction in this receptor's isoform levels in endometrial tissue [17]. Moreover, hypermethylation of *HOXA10 *causes its reduced expression, accompanied with some defects in blastocyst implantation in mid-luteal endometrium [16].

We observed that decreased *HOXA11 *expression was associated with hypermethylation of *HOXA11 *CpG rich regions in eutopic mid-secretory endometrium from infertile women with endometriosis compared to fertile women. Our findings may support a view of endometriosis as an epigenetic disease.

HOXA11 protein alone is a repressor of the decidual prolactin promoter, but combined with FOXO1A transcription factor induces transcription of decidual prolactin [47]. Therefore, *HOXA11 *expression may control the proper production of decidual prolactin, which is essential for implantation and pregnancy [48]. This may indicate that changes in *HOXA11 *expression in eutopic endometrium throughout the implantation window can be one of the possible molecular mechanisms of endometriosis-associated infertility in women.

## Competing interests

The authors declare that they have no competing interests.

## Authors' contributions

MS contributed to designing the study, acquisition of data, analysis and interpretation of data, and in writing the manuscript. PW participated in the in the acquisition and interpretation of the data. As Principal Investigators, JS and JPP were involved in the intellectual and experimental programming of the study, the assays and interpretation of data, and writing the manuscript. All authors read and approved the final manuscript.

## Supplementary Material

Additional file 1 **Supplemental Table S1**. Primer sequences used for RQ-PCR analysis and bisulfite sequencing of *HOXA11 *regions I, II, and III. Click here for file

Additional file 2 **Supplemental Figure S1**. Location of CpG-rich regions I, II, and III in the human *HOXA11 *gene. Click here for file
