# Supplementary material for: Expression of HOXA11 in the mid-luteal endometrium from women with endometriosis-associated infertility
Source: Reprod Biol Endocrinol. 2012 Jan 10;10:1. doi: 10.1186/1477-7827-10-1 (PMC3275521; doi:10.1186/1477-7827-10-1)
Supplement: Additional file 1 — Supplemental Table S1. Primer sequences used for RQ-PCR analysis and bisulfite sequencing of HOXA11 regions I, II, and III. [file 1477-7827-10-1-S1.PDF]

Table S1. Primer sequences used for RQ-PCR analysis and bisulfite sequencing of *HOXA11* regions I, II, and III.

Gene	Sequence (5'-3' direction)	Position	ENST number www. ensembl.org/	Product size (bp)
HOXA11	CTCAGTGTCTGGCTGCAGAG	+2623 ; +2643	00000006015	136
	GCTTCCAAGCTCAGTTCAAGA	+2738 ; +2756		
ACTB	GCACCACACCTTCTACAATGAGC	+450 ; +473	00000331789	166
	GGATAGCACAGCCTGGATAGCAAC	+592 ; +616		
GAPDH	TGCCAAATATGATGACATCAAGAA	+1422 ; +1446	00000229239	121
	GGAGTGGGTGTCGCTGTTG	+1524 ; +1543		
DNMT1	TACCTGGACGACCCTGACCTC	+1255 ; +1275	00000130816	103
	CGTTGGCATCAAAGATGGACA	+1336 ; +1358		
DNMT3A	TATTGATGAGCGCACAAGAGAGC	+1648; +1670	00000119772	111
	GGGTGTTCCAGGGTAACATTGAG	+1736; +1759		
DNMT3B	GGCAAGTTCTCCGAGGTCTCTG	+1119 ; +1141	00000088305	113
	TGGTACATGGCTTTTCGATAGGA	+1209 ; +1232		
HOXA11(I)	GAGGATATGGGAGGTAGTGG	-2856 ; -2837	00000006015	449
	CCAAACTCTCTCTCTACAACC	-2428 ; -2408		
HOXA11(II)	GTTAAGGATGGGGATAGAT	-63 ; -45	00000006015	386
	AAATAACAATAAACCAAATTAC	+302 ; +323		
HOXA11(III)	AAGGTTTATGAAGGGTTTTTAGG	+880 ; +902	00000006015	615
	AACAAACTCTACTTACTCCCC	+1474 ; +1494		

The position of the primers was counted from the first nucleotide encoding HOXA11, ACTB, and GAPDH, DNMT1, DNMT3A, DNMT3B transcript. *HOXA11I-III* correspond to primers complementary to the bisulfite-DNA modified in *HOXA11* regions I, II, and III (Figure 1S).
