# Supplementary material for: Expression of HOXA11 in the mid-luteal endometrium from women with endometriosis-associated infertility
Source: Reprod Biol Endocrinol. 2012 Jan 10;10:1. doi: 10.1186/1477-7827-10-1 (PMC3275521; doi:10.1186/1477-7827-10-1)
Supplement: Additional file 2 — Supplemental Figure S1. Location of CpG-rich regions I, II, and III in the human HOXA11 gene. [file 1477-7827-10-1-S2.PDF]

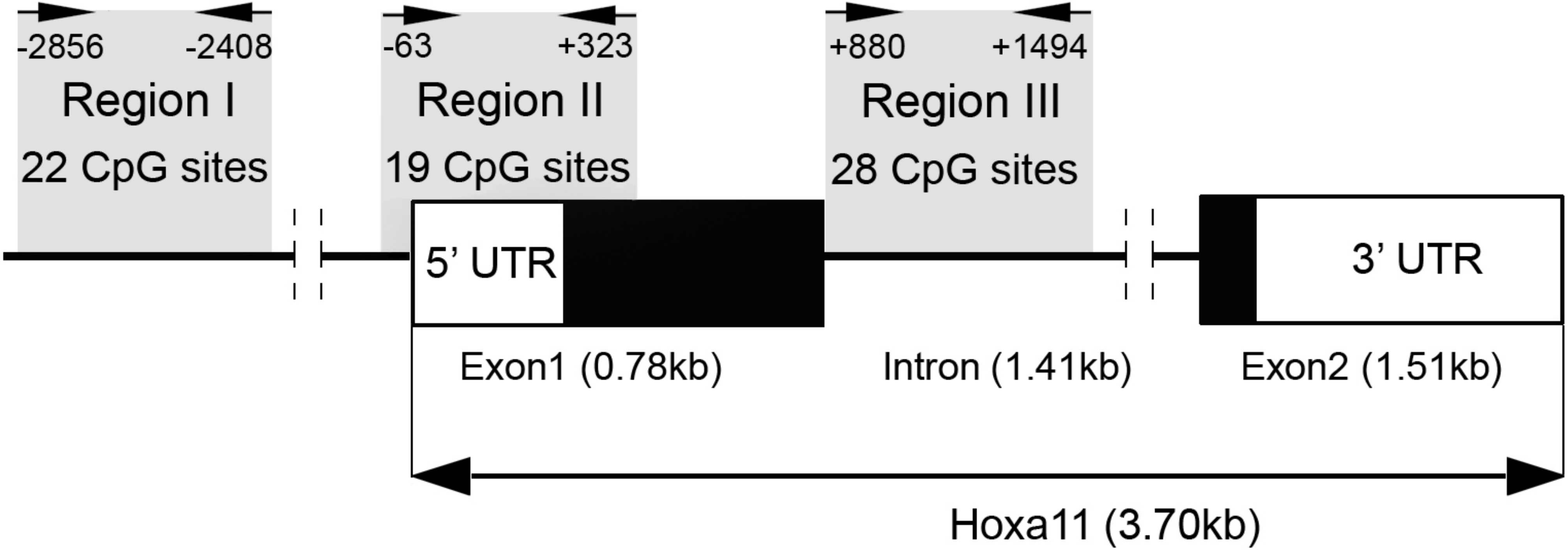

Figure S1. Location of CpG-rich regions I, II, and III in the human *HOXA11* gene

Three CpG-rich regions in the *HOXA11* gene were located: the first one (I, 22CpG) 2408 bp upstream of exon 1, the second (II, 19 CpG) mainly in the first exon, and the third (III, 28CpG) in the intron separating exons 1 and 2. The arrows correspond to the position of primers exploited for bisulfite sequencing of *HOXA11* regions I-II-III (Additional file 1, Table S1).
